# Humor Assessment and Interventions in Palliative Care: A Systematic Review

**DOI:** 10.3389/fpsyg.2018.00890

**Published:** 2018-06-19

**Authors:** Lisa M. Linge-Dahl, Sonja Heintz, Willibald Ruch, Lukas Radbruch

**Affiliations:** ^1^Department of Palliative Medicine, University of Bonn, Bonn, Germany; ^2^Personality and Assessment, Department of Psychology, University of Zurich, Zurich, Switzerland; ^3^Center for Palliative Care, Malteser Hospital Seliger Gerhard Bonn/Rhein-Sieg, Bonn, Germany

**Keywords:** humor, intervention, palliative care, end-of-life, systematic review

## Abstract

**Background:** The central goal of palliative care is to optimize the quality of life of patients suffering from life-limiting illnesses, which includes psychosocial and spiritual wellbeing. Research has demonstrated positive correlations between humor and laughter with life satisfaction and other aspects of wellbeing, and physiological symptoms can be improved by humorous stimuli.

**Objectives:** The aim of this review is to evaluate humor interventions and assessments that have been applied in palliative care and to derive implications for future research.

**Methods:** A systematic review of four databases identified 13 included studies. Criteria for inclusion were peer-reviewed English-language studies on humor interventions or assessments in a palliative care context.

**Results:** Two studies on humor interventions and 11 studies on humor assessment were included in the systematic review. Most of these studies were about the patients' perspective on humor in palliative care. Findings showed that humor had a positive effect on patients, their relatives, and professional caregivers. Humor was widely perceived as appropriate and seen as beneficial to care in all studies.

**Conclusions:** Even though humor interventions seem to be potentially useful in palliative care, descriptions evaluating their use are scarce. Overall, research on humor assessment and interventions in palliative care has remained limited in terms of quantity and quality. More research activities are needed to build a solid empirical foundation for implementing humor and laughter as part of regular palliative care activities.

## Introduction

### Rationale

Humor has been subject to research and philosophical reflections for centuries and has also been used for interventions in the health sector (Hulse, 1994). Most research has been conducted in pediatrics (review by Sridharan and Sivaramakrishnan, 2016). Apart from the health sector, humor interventions have also been investigated in the field of positive psychology (Ruch and McGhee, 2014; Ruch and Hofmann, 2017). Some studies in medical settings were conducted with older people in nursing homes (Mathieu, 2008; Goodenough et al., 2012; Low et al., 2013), cancer patients (Itami, 2000; Venter et al., 2008), veterans (Steinhauser et al., 2000), and patients suffering from depression (Shahidi et al., 2011). Positive correlations have been reported on humor and laughter in relation to life satisfaction outside the health care setting (Wild et al., 2003; Ruch et al., 2010), and there is some evidence of a relationship between humor and health (Martin, 2001, 2004).

The theoretical model of the effect of humor on health has been described by Martin (2008) and Gremigni (2012) extensively, who concluded that humor as a complex psychological phenomenon needs to be differentiated according to the kind of humor and the setting. Hearty laughter, for example, works through different mechanisms than social and interpersonal aspects of humor and results in different effects. Social and interpersonal aspects of humor, such as enhancing personal connections, influence health and wellbeing by increasing one's level of social support, while hearty laughter may predominantly affect health by improving the respiratory, musculoskeletal, vocal, and cardiovascular activity. Each kind of humor requires a specific research setting and will produce specific effects (Martin, 2008).

Society perceives humor to have beneficial effects on health and wellbeing (Boyle and Joss-Reid, 2004). Implementation concepts of humor and the scientific evaluation of their effects (Boyle and Joss-Reid, 2004) have been developed over the last century. These different kinds of interventions range from individualized humor therapy visits via the presentation of humorous movies aligned with patients' humor preferences (Schwartz and Saunders, 2010) to clowns working in the public health sector. Warren and Spitzer (2011) provided a summary of different types of clowns working in health care settings (e.g., elder-clowns and “classical” clowns in hospitals) in various countries and concluded that the application in elder and end-of-life care may not only benefit residents and patients, but health care professionals and family members as well. There are not only different types of clowns in healthcare but also different styles of humor that can be assessed (Craik et al., 1996; Schultes, 1997; Martin et al., 2003; Ruch et al., 2018). One of the few randomized controlled studies on humor interventions with adequate power was carried out in Australia and included 398 residents from nursing homes (Goodenough et al., 2012; Low et al., 2013). The single-blind randomized controlled study evaluated a clown intervention over a period of 9–12 weeks, which showed a significant decrease in agitation in residents compared to the control group receiving usual care. Additionally, so called “LaughterBosses” (staff members in nursing homes) were trained as facilitators with techniques to incorporate humor in between elder-clown visits. Humor also seems to be a relevant coping mechanism in various aspects of patients' lives. In her analysis of posts in an online patient-to-patient cancer forum, Demjén (2016) found that patients make fun of cancer and its consequences in multiple and creative ways to cope with their physical and psychological distress.

Despite these beneficial effects, there has been limited research on humor interventions for patients at the end of life. This might result from the societal perception that death is not supposed to be the object of implementations that included humor (Herth, 1990). Also, certain situations or topics might limit or impede the use of humor; for example, unfamiliarity between the patient and the health care professional (Erdman, 1991) or the fear of ridicule in certain patient groups, such as penile cancer patients (Branney et al., 2014).

However, the limited number of existing studies imply that humor might be beneficial toward the end of life as well (Steinhauser et al., 2000). Cox (1998) explored the effect of humor, art, and music on dying children through a literature review and found that any kind of social support and artistic strategies to process emotions and grief helps children: “[…] to remove the distance to others, find relief for depression, enhance their self-esteem, lower anxiety, fear and other feelings of grief and achieve an improved level of acceptance of reality” (Cox, 1998, p. 416). Cancer patients talk about humor as one of the predominant themes and coping strategies in their lives (Venter et al., 2008). Dean (1997) extrapolated findings from humor research in other health care settings and concluded that humor may be applied in the palliative care setting as well. However, she also noted that in certain situations, like crises and imminent death, humor would not be appropriate. From the perspective of health care professionals, Müller et al. (2012) found that humor is one of the three most powerful resources that protect health care teams from the negative effects of the strain of death and dying.

Kanninen (1998) conducted a review on humor in palliative care, but found only one pilot study that analyzed the effect of humor on 14 patients (Herth, 1990). The remaining articles included in Kanninen's review were anecdotal personal experiences of individuals. Kanninen concluded that research is needed to establish if humor is effective in medicine, especially in palliative care. The present paper reviews the study of Herth (1990) and the research that has been added in the two decades since Kanninen's review. It thus lays the foundation for future research on humor interventions in palliative care, assessing the effects on patients, relatives, and health care professionals.

### Objectives

The aim of this review is to synthesize humor interventions and assessments that have been applied in palliative care and to derive implications for future research and applications. The investigated patients were diagnosed with an incurable disease and were at the end of their lives. Study designs and outcomes of interventions and assessment are compared and grouped to facilitate cross-study comparisons.

### Research questions

This systematic review evaluates the effectiveness of humor interventions in a palliative care setting. It also outlines which kinds of humor interventions and assessments have been applied in palliative care until now and the methods, results, and limitations of these studies.

## Methods

### Study design

A systematic literature review of qualitative and quantitative research was undertaken in July 2017.

### Participants and interventions

The target group in the reviewed studies consisted of patients in a palliative care setting who received a humor intervention. Studies assessing the perspective of family caregivers or health care professionals on humor were also included. Different kinds of interventions and assessments were reviewed in a range of patient groups and institutions. All patients had diagnoses of incurable diseases and received end-of-life care.

### Systematic review protocol

**Figure 1:**
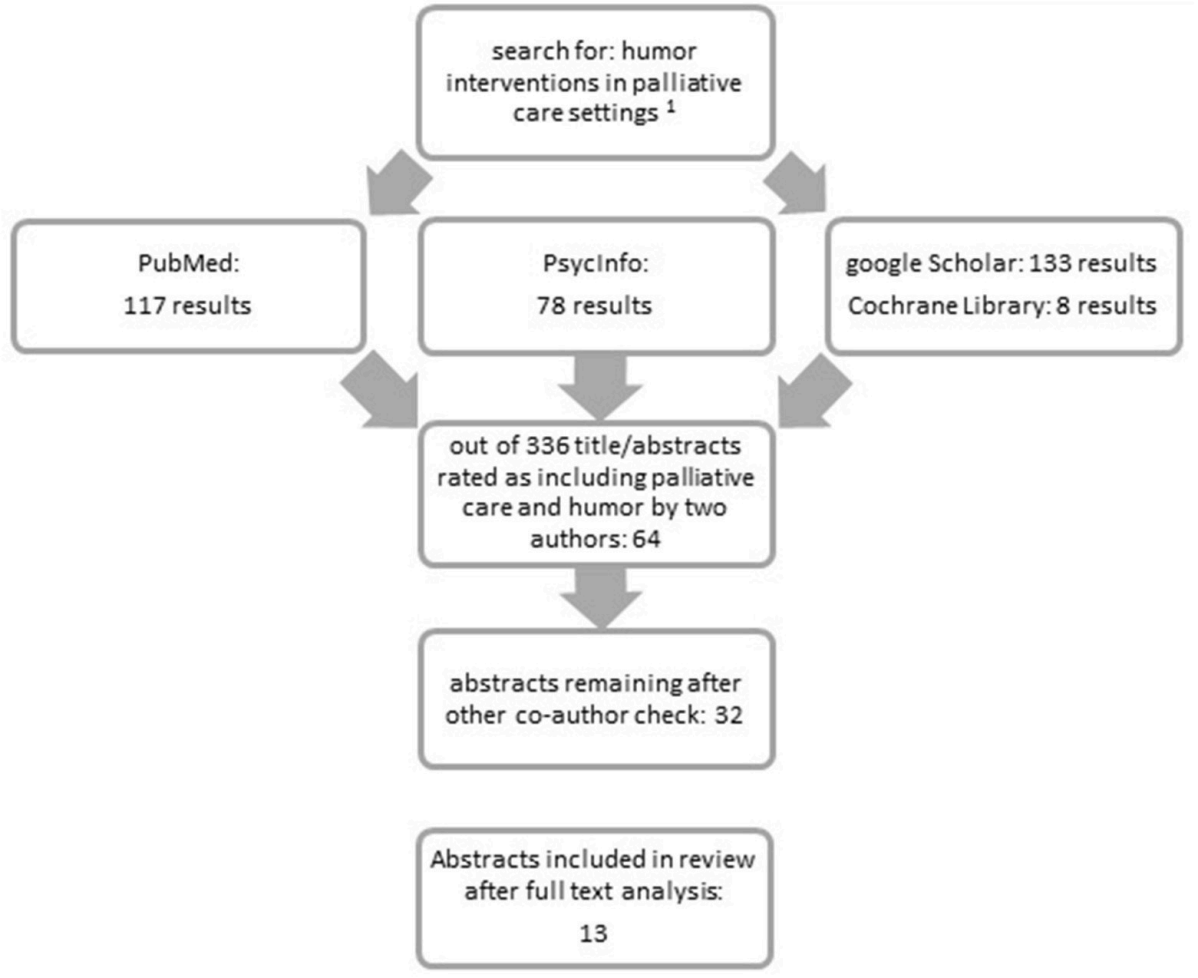
Flowchart of the review protocol. ^1^search terms: {(humor OR humor OR humorous OR clowns OR clown[Title/Abstract]) AND intervention OR training OR coaching OR visit OR practice OR therapy[Title/Abstract]} AND “palliative care” OR “hospice care” OR “end-of-life” OR geriatric OR “life limiting illness” OR death OR dying[Title/Abstract].

Overall, 336 abstracts were found and reviewed by two authors (LLD and LR), with an agreement rate of >95% regarding the investigated publications. Screenings resulted in 64 abstracts that were rated as potentially relevant for the review. Lack of consensus about inclusion was discussed with another author (SH). Next, 32 articles were analyzed as full-text versions, from which 13 met the inclusion criteria (see Figure 1), for further information please access the Supplementary Material. The included studies were published between 1990 and 2017. No older studies have been identified in the literature search. The 17 articles which were not included were an opinion paper (Dean, 1997) or articles that investigated patient groups which did not meet the criteria of palliative care (e.g., Low et al., 2015).

### Search strategy

Three search strings on the topics of humor, intervention, and palliative care connected by Boolean operators were used. The search terms were: {(humor OR humor OR humorous OR clowns OR clown[Title/Abstract]) AND (intervention OR training OR coaching OR visit OR practice OR therapy[Title/Abstract]) AND (“palliative care” OR “hospice care” OR “end-of-life” OR geriatric OR “life limiting illness” OR death OR dying[Title/Abstract])}.

Publications were included if they were published in a peer-reviewed journal, contained original qualitative or quantitative data, applied and/or assessed a humor(ous) intervention, evaluated effects on patients or residents in nursing homes receiving palliative care, and were published in English. The year of the publication of the study was not restricted.

### Data sources and data extraction

Four key databases (PsycInfo, PubMed, Google Scholar, and Cochrane Library of systematic reviews) were systematically searched to July 16th 2017. Full-text publications were downloaded via the library of the Medical Faculty of the University of Bonn.

### Data analysis

All included articles were reviewed in depth. The selected studies were divided into (a) studies that investigated humor in palliative care as the main goal of the paper and (b) studies in which humor emerged as an important variable from an initial research question that had not focused on this topic, for example assessing end-of-life wishes (Delgado-Guay et al., 2016). Target groups, participant numbers, publication bias, study methodology, and quality of research were also analyzed using a template. However, the wide range of different conceptualizations of humor in the studies as well as methodological weaknesses prevented meaningful comparison between studies. Results are presented according to target groups and study methodology. Effect sizes were analyzed using Cohen's (1992) guidelines. Potential bias within the studies was identified and discussed.

## Results

**Figure 2:**
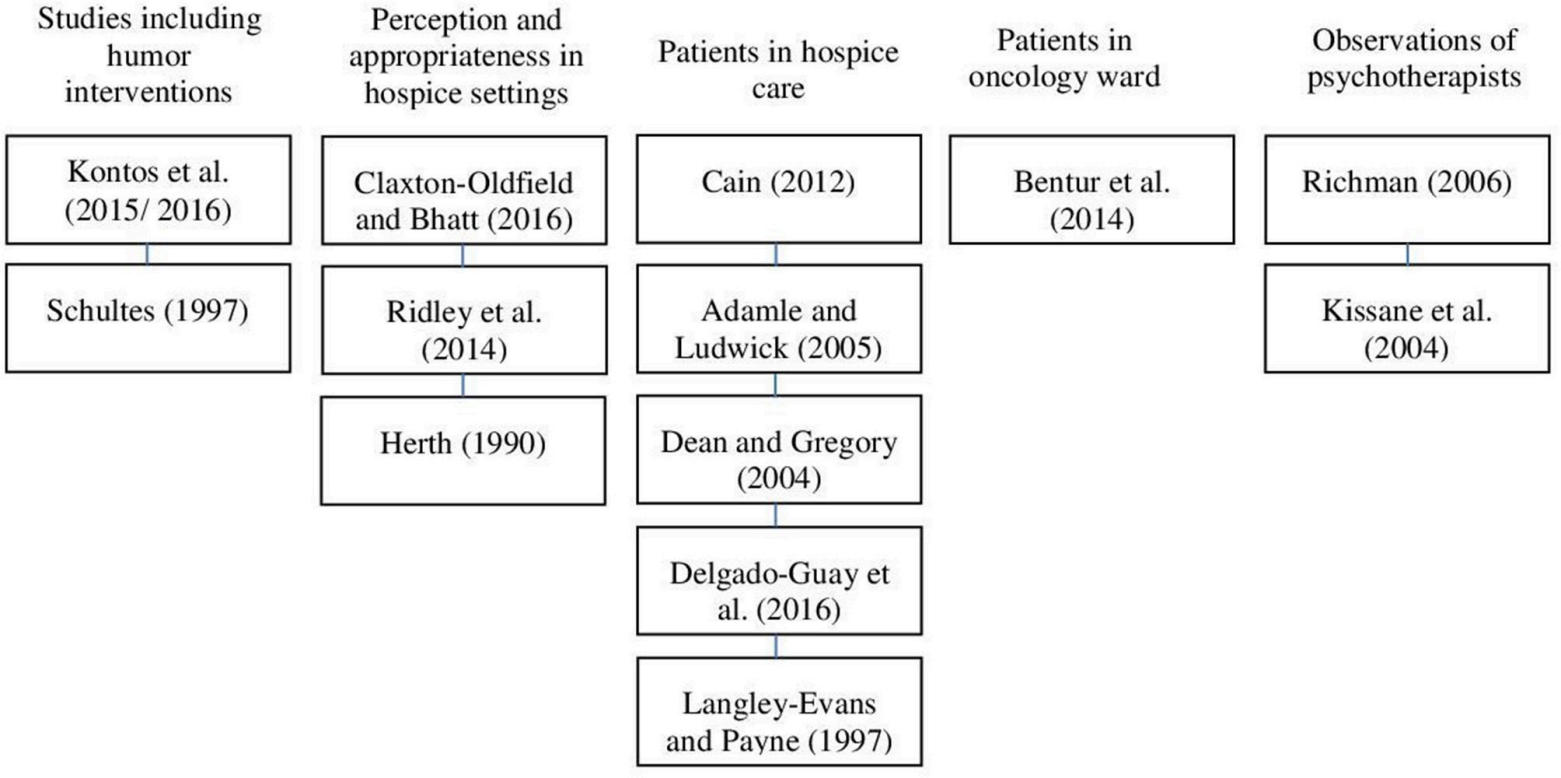
Flowchart of included studies.

The 14 included research papers contained data on 13 studies (see Figure 2). One study was published in two separate papers, one describing the qualitative results (Kontos et al., 2016) and the other discussing the quantitative results (Kontos et al., 2015). Ten articles were selected because they presented findings of interventions or assessments of humor as the main goal of the paper. Four other publications were included because they dealt with humor, among other variables, as a secondary outcome. Two publications focused on humor interventions and eight mainly on the assessment of patient's perception of humor, while three examined the perspective of caregivers and/or health care professionals. Nine publications described qualitative results (Herth, 1990; Langley-Evans and Payne, 1997; Schultes, 1997; Dean and Gregory, 2004; Adamle and Ludwick, 2005; Richman, 2006; Cain, 2012; Bentur et al., 2014; Kontos et al., 2015), and five articles presented quantitative results (Kissane et al., 2004; Ridley et al., 2014; Delgado-Guay et al., 2016; Kontos et al., 2016; Claxton-Oldfield and Bhatt, 2017). Overall, a total of 759 participants were included in the reviewed studies.

The results are presented in the following order: the two studies that included humor interventions (Schultes, 1997; Kontos et al., 2015, 2016), three studies exploring perception and appropriateness of humor in hospice settings (Herth, 1990; Ridley et al., 2014; Claxton-Oldfield and Bhatt, 2017), followed by five publications that assessed functions and results of humor applications on patients in hospice care (Langley-Evans and Payne, 1997; Dean and Gregory, 2004; Adamle and Ludwick, 2005; Cain, 2012; Delgado-Guay et al., 2016) and one on patients in an oncology ward (Bentur et al., 2014), followed by two studies presenting results from psychotherapists' observations (Kissane et al., 2004; Richman, 2006). Within each of the subsections of the results, the studies are presented in the order of their publication date beginning with the most recent one. At the end of each section, the main information is condensed in a table.

### Studies that included humor interventions

Two studies investigated the effects of humor interventions in a palliative care setting (Schultes, 1997; Kontos et al., 2015, 2016), one for patients with advanced dementia in nursing homes, and one for patients being treated by a hospice service at home. Both studies applied humor interventions in a palliative care setting. While one study used clowns (Kontos et al., 2015, 2016), the other study involved nurses using humor with the patient (Schultes, 1997). The outcome measures and the study participants varied strongly, limiting comparability between studies (see Table 1).

**Table 1:** Studies including humor interventions.

**Authors (year of publication)**	**Target group (N)**	**Intervention/assessment**	**Main results**
Kontos et al., 2015, 2016	Nursing home residents (23)	Elder-clown interventions	Humor interventions of elder-clowns resulted in a decreased level of agitation and a nominal decrease in dosing of psychotropic medications. Observation protocols showed improvements in the patients' expression of joy, sadness, reciprocal play, and co-constructed imagination.
Schultes, 1997	Patients receiving home-care or in hospices (1/case study)	Humor intervention implemented and evaluated by hospice home-care nurses	The case study showed that the patient responded well to the humorous films and a change of the mood was perceived from the family as well. The film material was even used after the patient's death and helped the relatives to cope with the loss.

A Canadian study using so called “elder-clowns” (with a red nose, but minimal make-up and clothing from an earlier era) applied approximately 10 min humor interventions twice a week over a period of 12 weeks to nursing home residents in an advanced stage of dementia (Kontos et al., 2016). No control group was investigated, so bias cannot be ruled out. The qualitative results of the study were published separately (Kontos et al., 2015). The clowns used improvisations, humor, empathy, song, musical instruments, and dance. Data collection involved video recording the interventions, and the clowns were interviewed afterwards. Several researchers screened and transcribed the videos to assure interrater reliability. The aim of the intervention was to achieve “relational presence,” a term that Kontos et al. define as: “[…] the reciprocal nature of engagement during plays, and the capacity of residents to initiate as well as respond to […] creative engagement” (p. 5). To facilitate an appropriately tailored intervention for each participant, so called “census information”—personal preferences, history of the patient and personality—was informally collected from staff or family. With a small number of participants (*N* = 23) a significant improvement was found between the baseline and the end of intervention scores in “behavioral and psychological symptoms of dementia” (from *M* = 24.4; *SD* = 12.9 baseline to *M* = 18.6; *SD* = 13.1 after 12 weeks; scale from 0 to 144; *t* = −2.68, *p* = 0.01; Cohen's *d* = −0.45), quality-of-life (from *M* = 0.04; *SD* = 0.51 baseline to *M* = 1.05; *SD* = 0.29; scale from −5 to 5; after 12 weeks; *F* = 23.09, *p* < 0.001; Cohen's *d* = 2.44) and “occupational disruptiveness” (from *M* = 8.09; *SD* = 7.1 baseline to *M* = 4.9; *SD* = 5.2 after 12 weeks; scale from 0 to 60; *t* = −2.58, *p* = 0.02; Cohen's *d* = −0.51) using questionnaires completed by the nursing staff and family members. Use of psychiatric medication and nursing burden did not change significantly. There was a tendency for decreased agitation/aggression, but this did not reach statistical significance (from *M* = 3.3; *SD* = 3.3 baseline to *M* = 2.1; *SD* = 2.0; scale from 0 to 12; *t* = −1.86, *p* = 0.07; Cohen's *d* = −0.44). The authors report that persons diagnosed with dementia could engage in the humor interventions in different ways even though they were in their last stage of life. This engagement ranged from sharing their sadness to reciprocal play, joy, imaginative exploration, and from recognizing humor to even creating humor on their own initiative.

The second intervention was developed after an analysis of the existing literature on humor in health care. Schultes (1997) evaluated a humor intervention for patients treated by hospice home care nurses. The intervention was guided by humor assessment questions to explore the preferred style of humor (e.g., incongruity, nonsense, ridicule, or slapstick) and instructions for nurses on how to observe humorous behavior. After the assessment procedure, humorous cassettes and movies were shown to the patient according to the preferred humor style. The intervention was tested in a clinical case study with a 65-year-old woman suffering from metastatic colon cancer. Data collection on the intervention effects was based on observations of the patient by nurses and informal interviews with the patient's relatives. The results of the case study indicated that humorous interactions and listening to humorous cassettes or watching funny movies made the patient feel better, that she demanded less pain medication and smiled more, and that it also improved the quality of her remaining life. Even after the patient's death, her family reported that they continued to watch the movies, which helped them to feel relieved and to cope better with their grief, and which gave them a sense of power in a situation where they felt weak. However, the authors did not follow up the case report with a humor intervention trial and the lack of an independent researcher in the data collection could have led to biases.

### Studies assessing or observing humor in an explorative way

#### Exploring perception and appropriateness in hospice settings

Three studies assessed the appropriateness of humor as an intervention in hospice settings using qualitative data (Herth, 1990), quantitative data in general (Ridley et al., 2014), and quantitative data on volunteer-patient interactions (Claxton-Oldfield and Bhatt, 2017). Humor was perceived as appropriate or even essential in those settings, though the authors mentioned limitations regarding the use of humor, such as impending death or absence of a sense of humor (see Table 2).

**Table 2:** Studies exploring perceptions and appropriateness in hospice settings.

**Authors (year of publication)**	**Target group (N)**	**Intervention/assessment**	**Main results**
Claxton-Oldfield and Bhatt, 2017	Volunteers working in hospice or palliative care (32)	Humor application during patient visits	Humor was applied after getting to know the patient and following the patients lead (40.7%), depending on his stage of illness (41.4%). 96% of the volunteers stated that there is a place for humor in palliative care and 88.9% stated that humor helped them to cope with the demands of their voluntary work.
Ridley et al., 2014	Patients in palliative care units or residential hospices (100)	Questionnaire about appropriateness of humor in palliative care	Patients valued humor as important prior (77%) and during (76%) their illness. The frequency of laughter declined from 65% of patients who laughed 16 or more times a day prior to the illness to 22% during the illness.
Herth, 1990	Terminally ill adults (14)	Structured interview to explore patients views on humor	Eight participants reported humor to be an important part of their lives prior to the illness. Twelve stated that humor would be helpful in the present situation, but only two indicated a presence of humor in their lives.

Herth's (1990) small study on 14 terminally ill adults receiving hospice care at home explored patients' perceptions of and experiences with humor in structured interviews. Patients explained that humor incorporated the following improvements: connectedness, change of perspective, hope, joy and relaxation including physiological improvements. Also, the majority (12 of the 14 participants) of the interviewees stated a need for humor, indicated by quotes such as “Everyone is so sad,” “It just makes it harder, I wish we could lighten up,” and “If I ever needed humor it is now” (Herth, 1990, p. 38). The author concluded that terminally ill people appeared to be the ones who benefitted the most from humor interventions. As a coping mechanism, humor becomes essential due to deteriorating body functions, unfamiliar procedures, and physical and emotional suffering. Humor was also described as one of the most powerful coping mechanisms. However, the strong conclusions that the authors drew may be questioned in relation to the small sample size of the study.

Ridley et al. (2014) analyzed whether humor is appropriate in a palliative care setting. They interviewed 100 patients in palliative care units and residential hospices. A standardized questionnaire captured patients' perception toward humor therapy prior to and during their illness (Ridley et al., 2014). Ridley et al. reported a potential “bias inherent to retrospective self-reporting” (2014, p. 474). Most participants valued humor as important prior to (77%) and during (76%) their illness. However, the frequency of laughter in patients who laughed 16 or more times a day declined from 65% prior to the illness to 22% during the illness. Patients who rated humor to be more important than other patients were more likely to consider themselves as funny before (*p* < 0.001) and during (*p* = 0.014) their illness.

The perception of appropriateness, types, frequency, and results of humorous interactions in hospice and palliative care patients during their interaction with volunteers was analyzed by Claxton-Oldfield and Bhatt (2017) from a volunteers' (*N* = 32) point of view. A quantitative questionnaire was developed on the basis of an informal discussion with four volunteers. The first part of the questionnaire examined the frequency of humor in patient-volunteer interactions (for example “How often do your patients initiate humor with you during your interactions with them?”). The second part examined the acceptability of humor in interactions. The volunteers visited patients in a range of different settings (hospital, client's home, nursing home, and residential hospice). The authors report a potential bias from nonresponse. More than half of the volunteers rated humor as very or extremely important in interactions with patients. In most cases humor was applied (a) after getting to know the patient and following the patients' lead (*n* = 11; 40.7%) and (b) depending on his/her stage of illness (*n* = 12; 41.4%). Impending death was perceived as a very inappropriate moment for the use of humor. All in all, 96% (*n* = 31) of the volunteers believed that there was a place for humor in palliative care, and 88.9% (*n* = 24) stated that humor helped them to cope with the demands of their voluntary work.

#### Assessment of functions and results of humor application

##### Patients in hospice care

Five studies examined the functions and results of humor applications (see Table 3). All of them used observations and interviews as methods of data collection. The results demonstrated that humor was crucial for hospice professionals to cope with the demands of their jobs (Cain, 2012), that it was primarily initiated by patients (Adamle and Ludwick, 2005), and that it helped health care professionals and patients to build relationships and to bear difficult situations. Humor was, moreover, a means to express sensibility (Dean and Gregory, 2004), it represented an important end-of-life wish (Delgado-Guay et al., 2016), and it helped patients to distance themselves from their own death (Langley-Evans and Payne, 1997).

**Table 3:** Studies assessing functions and results of humor application.

**Authors (year of publication)**	**Target group (N)**	**Intervention/assessment**	**Main results**
Delgado-Guay et al., 2016	Advanced cancer patients (100)	Assessment of end-of-life wishes	45% of the participants rated “to keep my sense of humor” as one of the ten most important end-of-life wishes.
Cain, 2012	Hospice professionals (41 + 7 informal interviews)	Participating observation, structured and informal interviews	Humor was found to inherit an important role predominantly in the back region of the hospice. Humor was found as an instrument of distancing, to enable professionals to deal with emotional difficult times, and being a resource of strength to continue their job.
Adamle and Ludwick, 2005	Patients in hospice care (160)	Observation of patient-nurse –primary caregiver interactions	Humor was observed during more than 80% of all observed visits. 70% of this humor was initiated by the patients, 17% by the nurses, and 12% by the caregivers. The average number of humorous remarks per visit was three.
Dean and Gregory, 2004	Patients, their families and health care professionals in palliative care (15 interviews)	Participating observation, semi-structured (health care professional) and informal interviews (patients and family)	Humor was found to be pervasive and persistent in palliative inpatient care. Three main functional categories of humor emerged from the data: building relationships, contending with circumstances, and expressing sensibility.
Langley-Evans and Payne, 1997	Patients in a day care palliative care ward (14)	Participant observation and informal interviews	Rather nonverbal humorous nature in this “death talk” enabled the patients to distance themselves from their deaths.

Delgado-Guay et al. (2016) compared four different tools developed to rate end-of-life wishes in a randomized controlled trial (RCT). Hundred patients with advanced cancer in an inpatient palliative care unit in South Texas rated “to keep my sense of humor” as one of the ten most important end-of-life wishes (45% of all participants).

Cain (2012) analyzed “front” and “back region” personalities of health care professionals; that is, the personality shown in front of patients and relatives on the one hand, and the personality presented in team meetings and with colleagues on the other hand. Data was collected through observations over 1 year by a researcher at the ward and 51 interviews with staff-members. Bias was possible because only one researcher collected the data, so no inter-rater checks were conducted. Among the dynamic and complex interactions of staff and patients, she found that humor fulfilled an important role, predominantly in the “back region” of the hospice staff. It was not only an instrument to distance oneself from negative emotions, but also a resource of strength, which enabled professionals to deal with emotionally difficult times.

Adamle and Ludwick (2005) observed 132 interactions between nurse, patient, and primary caregivers in hospice settings (home care hospice services, inpatient hospice, and hospice care in nursing homes) including 160 participants. They counted the number of occurrences of humor and who initiated them. Potential bias was reported in the selection process of participants. In three different settings, humor was observed in 85% of the 132 observed interactions. In about 70% of the cases, humor was initiated by the patient, and the average number of humor occurrences per visit was three. The lack of humorous occurrences in 15% (*n* = 20) of the observed interactions was due to the cognitive inability or impending death of the patient (nine patients were either in a coma or did not respond physically or mentally to verbal cues, and five patients were dying).

In another study, Dean and Gregory (2004) focused on the circumstances, functions, and appropriateness of humor in an inpatient palliative care unit using participant observation plus informal and structured interviews with 15 health care professionals. Detailed field notes and transcribed interviews were analyzed. Humor was reported to be “pervasive and persistent” (p. 140) and had the following key functions: (a) building relationships (making connections, humor as attraction, discovering hidden verbal messages, energizing, nurturing community, neutralizing status differences), (b) bearing the situation (humor as respite, humor as survival, humor as tension relief/lightening the heaviness, maintaining perspective/providing support), and (c) expressing sensibility (preserving dignity, acknowledging personhood).

In their ethnographic investigation, Langley-Evans and Payne (1997) studied how patients in a palliative day care unit think and talk about their condition and death, using participant observation over a period of 7 weeks and evaluating field notes and documentary information from health care professionals. One theme that emerged from the qualitative data analyses was the rather nonverbal humorous nature of this “death talk,” which enabled patients to distance themselves from their own deaths.

### Patients in other settings

Three studies examined patients in other settings (see Table 4). Bentur et al. (2014) analyzed coping strategies at the end of life in 22 advanced cancer patients in an Israeli daycare oncology clinic. The interviews were transcribed verbatim and analyzed afterwards. Humor was described as one of the five applied coping strategies. One participant stated on the use of humor “maybe it helped me ease the burden” (Bentur et al., 2014, p. 4).

**Table 4:** Studies with patients in other care settings.

**Authors (year of publication)**	**Target group (N)**	**Intervention/assessment**	**Main results**
Bentur et al., 2014	Advanced cancer patients (22)	In-depth interviews about coping strategies	Humor was found to be one of five coping strategies that were applied by the patients.
Richman, 2006	Patients receiving psychotherapy (8)	Investigation during psychotherapy	Ten features of humor in psychotherapy with patients at the end of their lives emerged. This included empathy, connectedness, the possibility to mentally distance from death, and the reduction of stress.
Kissane et al., 2004	Women with advanced breast cancer (227)	Supportive expressive group therapy	Amongst other topics, genuine humor was found to be a sign of a healthy functioning group in group therapy.

Two studies in a psychotherapeutic setting with end-of-life care patients extracted data from participant (therapist) observations and showed humor as an unplanned result of an explorative observation. Richman (2006) discussed the functions of humor in psychotherapy. Ten features of humor were developed by Richman based on eight patients, at the end of their lives, receiving psychotherapy. There is a risk of bias due to an unclear selection process of the patients. Skills in the use of humor were found to be necessary for psychotherapists treating patients at the ends of their lives or facing the topic of death. The ten features of humor were: (1) emerges spontaneously, (2) timing is essential, (3) fosters social cohesion, (4) power to reduce stress, (5) enforces feeling of community, (6) permits to distance from death, (7) the content of humor can be negative, (8) communication is essential, (9) requires a healing therapist with empathy, and (10) feeling of commonness.

In a large RCT study on 227 women with metastatic breast cancer, the topics and facilitating aspects of a weekly supportive-expressive group therapy were qualitatively analyzed (Kissane et al., 2004), indicating that genuine humor was a sign of a healthy functioning group. Furthermore, notes of the co-therapists were cross-checked by the main therapists and analyzed qualitatively, resulting in five categories: (1) the structure of supportive-expressive group therapy, (2) the role of therapists, (3) key themes, (4) group transformation, and (5) anti-group phenomena.

## Discussion

### Summary of main findings

By systematically reviewing the state of the art of humor in palliative care two decades after the review of Kanninen (1998), which included only one study on a humor intervention, we were able to include 13 studies in this review. Study results suggested that humor is an appropriate and useful resource in palliative care, but only two studies evaluated humor interventions in palliative care, and only one of the two was a RCT. Most of the reviewed publications explored and observed humor in different settings. There was no consensus on a definition of humor, on types of intervention, or on the assessment of effects that would allow comparisons of the published trials. Thus, studies were difficult to compare due to a different understanding of what humor interventions should look like, what they should accomplish, and which group of professionals should implement these interventions. Still, some conclusions about the benefits of humor can be derived from the reviewed studies.

One of the key benefits of humor in health care, which was reported in several trials, was an increased pain tolerance (Weisenberg et al., 1995; Zweyer et al., 2004). This finding was also in line with Herth's study (1990) in a palliative setting. However, it needs to be stated that RCT studies would be necessary to show whether the increase in pain tolerance (cold pressure test) was really due to the humorous stimuli or related to distraction or other factors.

Konradt et al. (2012) demonstrated the effect of a humor therapy group on older patients suffering from depression, which led to lower levels of seriousness and higher satisfaction with life scores in comparison to the control group. The study by Kontos et al. (2015) also highlighted the positive impact of clown interventions on physical and psychological well-being, demonstrating the benefits of the holistic approach. These statements need to be interpreted very carefully in relation to the small sample sizes that have been examined. The SMILES model for the implementation of humor in palliative care (Borod, 2006) was developed on the basis of a literature review about uses of humor and was modeled on the SPIKES model for the delivery of bad news in health care (Baile et al., 2000). SMILES aims at facilitating the use of humor in patient-physician interactions. The categories of this model are “**s**mile” (enter patient room with a smile), “**m**ake eye contact” (look and actually see the patient), “**i**ntuition and imagination” (sense appropriate cues for humor introduction), “**l**ook for, listen to, and Leap at the Opportunity” (get the real meaning of patients statements, so register subtle cues), “**e**lephants never forget” (remember exchanges with the patient and use them in following interactions) and “**s**ensitive to situation” (be aware of appropriateness of humor due to the situation). All these categories were illustrated by examples and aim at the application of humor in an appropriate and successful way. The success was not evaluated and bias in the selection of categories is possible.

But how does humor compare to other interventions in terms of well-being? Wellenzohn et al. (2016) tested the effect of different online humor interventions against a control group that reported early childhood memories and found humor to be efficacious. It needs to be noted though that this study included only healthy adults, and humor interventions would thus have to be tested in hospital patients at the end of their lives to provide conclusions for the target group of the present systematic review. Auerbach et al. (2016) were able to show that clinic clowns can induce more positive emotions than a circus clown and a nurse interaction by assessing the patients' current emotional state. Lacking in the literature is a comparison of humor interventions with other interventions such as music interventions, relaxation, yoga, or art therapy in palliative care (Koch et al., 2016). These controlled studies should include humor interventions as well as active control groups, including comparable interventions like music and art interventions and groups receiving usual care or additional nursing care to determine which beneficial effects are due to humor and laughter, and which ones are due to indirect factors (such as increased positive emotions or more interpersonal contacts). Future studies should also investigate whether humor interventions (in comparison to control groups) can lead to a decrease in the consumption of analgesics as well as a decrease in self-reported pain intensity. In addition, a longitudinal study setting would be preferable for future research as generalizations are limited for the results of cross-sectional studies.

However, there are discrepancies concerning the aim of the humor intervention. While Kontos et al. (2015) stressed that sadness and frustration need to receive sufficient attention and space, Schwartz and Saunders (2010) stated that the aim of humor therapy is to make patients laugh. Kontos et al. further emphasized that the aim of humor interventions is not to make the resident laugh, but to ease his/her state of mind and work with whatever is possible at that very moment. Similarly, the American Cancer Society (quoted in Schwartz and Saunders, 2010, p. 554) defined humor therapy as “[…] the relief of physical or emotional pain and stress and as a complementary method to promote health and cope with illness”. Apart from different definitions and concrete applications of humor, the consent of all investigated studies was that humor is not only valuable, but an important component of palliative care: “[…] humor is the glue that helps to put the connection together […] and as Palliative Care is all about relationships […] it would be incomplete” (Dean and Gregory, 2004, p. 141).

Not losing one's sense of humor was rated as an important spiritual end-of-life need (Delgado-Guay et al., 2016). These results might differ significantly in other cultural and spiritual settings, but we found no publications on the use of humor outside the Western-European cultural setting.

It has been stated that the sense of humor remains intact in people and even increases toward the end of life (Ruch et al., 2010). Thus, humor interventions are meaningful throughout the whole lifespan, including the end of life. Conducting humor interventions with patients in palliative care makes sense with the limitation that a sense of humor needs to be present in those individual patients taking part (Ruch and Hofmann, 2012; Auerbach, 2017), and the participants should not suffer from gelotophobia (the fear of being laughed at; Ruch et al., 2014).

There were several approaches to assess the patients' preferred kind of humor and whether they perceive humor as appropriate in their individual situation. Asking patients whether they consider themselves to be funny might be used as a screening question to identify people who find humor in their interactions with care providers appropriate (Ridley et al., 2014). However, humor production (being funny) is different from humor appreciation (perceiving humor as appropriate and helpful). Additionally, this kind of question needs to be used with care and considering the patient's actual emotional and spiritual situation. Adamle and Ludwick (2005) suggested that humor should occur without cues or prompting, enabling spontaneous humor. This would require an emotional atmosphere in the palliative care setting that allows the expression of humor from the patient's point of view. However, there were also critical voices that point to the use of off-color humor (gallows humor) amongst health care professionals (Piemonte, 2015). Self-disparagement related to functional defects was found to be predominant in elderly care, but should be initiated by the residents themselves, as otherwise it could be counterproductive (Keltner and Bonanno, 1997). To understand the benefits and limitations of the use of humor in palliative care, researchers need to conceptualize humor as a continuous rather than a binary concept (to have or not have a sense of humor), and they need to consider different facets of humor, ranging from benevolent humor to mockery (see Craik et al., 1996; Ruch et al., 2018). Both the “flavor” of humor (e.g., supportive, critical) as well as the targets (who jokes about whom) need to be taken into account, because it might heavily influence the impact of the use of humor. As a result, humor in palliative care settings should be social, benevolent, and supportive for the patient and his/her family.

The positive effects of humor on mourning relatives reported by Schultes (1997) has also been assessed by Keltner and Bonanno (1997) in a more structured way using questionnaires and structured interviews. However, family caregivers of patients receiving palliative care have not yet been included in a study in a structured and adequate way to comprehensively assess the effect of humor interventions with them.

In the field of professional caregivers and volunteers, humor was observed to be a valuable resource. Cain (2012) recorded statements of hospice workers saying that former colleagues, who quit their jobs because they could not handle the emotional burden, supposedly did so because they had lacked a sense of humor. This implies that humor is an ingredient to successful performance in this field (Müller et al., 2012). Measurement tools for assessing individual differences in humor could also be useful in the area of palliative care (for reviews see Ruch, 2007; Ruch et al., 2014). Critical aspects of humor such as sarcasm and cynicism could be potentially detrimental in the area of palliative care and thus need to be analyzed in more detail (Ruch et al., 2018). Importantly, assessing humor might put more strains on palliative patients (e.g., in terms of concentration, comprehension, and effort) than on healthy adults, for which humor measures were usually developed and tested. Thus, existing instruments might likely need to be adapted and pre-tested to ensure that the measurement is feasible and ethical in palliative patients. For example, short and/or simplified versions might need to be employed, or the items might need to be read to the patients. This need for short assessment tools has become clear in an unpublished pilot test of our research group.

Attrition numbers are an important component when analyzing the effects of humor interventions, because it is possible that certain people are more likely to remain in this kind of study setting. Low et al. (2013) reported a dropout of 16 residents from the initially 414 people that have been assessed for eligibility. Of those 16 residents, six did not give consent to participate in the study and 10 died or were transferred to a different location. Kontos et al. (2016) reported screening 45 residents, from which 23 were recruited. No information was provided on the selection process. The authors stated that during the intervention, 10 residents received all treatments, whereas 13 missed an average of 2.3 of the 24 visits. It needs to be taken into account that this kind of dropouts needs to be analyzed carefully in future research to explore potential differences in humor-related traits (such as gelotophobia or the sense of humor) of people who stay in humor intervention studies and those who drop out or decline to participate in the first place. Identification of potential responders might be difficult though, as data from people who decline to participate in a study usually is scarce. The study of Wellenzohn et al. (2016) gave detailed information on a 25% dropout rate from all four investigated groups. The dropouts were younger, with a predominance of men, yet they did not differ from other participants in their baseline levels of happiness or in depressive symptoms.

### Limitations

Our search strategy focused on publications in peer-reviewed journals and English language, and thus some interesting and potentially relevant results published in dissertations or in other languages could not be included. Overall, the search strategy might have been too restrictive with its focus on palliative care, as results from other areas of medicine might be transferred to the palliative care setting. However, the cognitive and physical impairment of patients with advanced life-limiting diseases and the high prevalence of depression in these patients put this comparability into question. It is also possible that studies have been published in nonmedical or psychological journals that were not included in the databases chosen for the present systematic review. However, any of these expansions would have gone beyond the scope of this paper.

The findings of the analyzed studies were often based on either self-reports or observations. To ensure the validity of the findings, multi-method studies, such as the study by Kontos et al. (2015, 2016), would be worthwhile. Ideally, these studies should combine for example self-reports, other-reports, physiological measures, and behavior observations, and they should include the perspectives of patients, caregivers, and health care professionals alike.

The small effect sizes of the quantitative studies need careful interpretation. Due to the small sample sizes, the effect sizes, according to Cohen's guidelines (1992), were not interpretable as representative results. Larger samples would be needed to demonstrate the efficiency of the interventions in the studies of Kontos et al. (2016), Claxton-Oldfield and Bhatt (2017), and Adamle and Ludwick (2005). Limitations of studies with small sample sizes (Ioannidis, 2005; Maxwell et al., 2008) also imply that for the study of Kontos et al. (2016) a careful calculation of sample size and power analysis would have been required to improve the quality of results. Using multiple comparisons (e.g., Kontos et al., 2016) would also require corrections for alpha error accumulation, if appropriate to the design (Armstrong, 2014).

The risk of bias has been assessed, and no bias has been found due to mutual cross-checks of the selection of articles between two authors. A publication bias may have affected the published literature because studies with significant positive results are more likely to be published than those without significant results.

A documentation template had been developed for our review, but with only scarce information on the quality of research and details on effect sizes, the scheme did not deliver usable results. A different template with a lower focus on study quality might have been more suitable. In general, the quality of the included studies was not as high as would have been desirable for a systematic review. RCTs of the field are needed. These should include humor interventions as well as other comparable interventions such as music and art interventions as well as a control group receiving usual care. Consensus should be sought for evaluating instruments and study settings for the different types of humor in order to provide meaningful data for comparisons and meta-analyses (Martin, 2008).

It needs to be noted that conducting research in palliative care settings needs to be designed with caution to avoid adding to the burden of patients and relatives with assessment and data collection. Also thorough coordination with nursing staff, physicians, relatives, other research staff and the patients themselves is crucial.

## Conclusion

The review of the literature has shown that 20 years after the first systematic review, there is still only limited research available on the use of humor interventions and assessments in palliative care. Researchers from different fields agree that humor is not only a valuable resource for patients, but also for health care professionals working with patients at the end of life. A few studies have looked at the effect of humor interventions in this group of patients, mostly with promising results. Still, improved quality of life, better communication and sense of connectedness to staff and family members, the ability to distance oneself from the problems and burdens of the illness, and sometimes enabling a decreased perception of pain have been demonstrated. However, there is no consensus on a definition of humor, on types of interventions, or on the best method to assess the effects that would allow comparisons between published trials. Clearly, more research on the use of humor in palliative care is needed. Advancements in outlining the field of humor (Craik et al., 1996; Ruch et al., 2018) and the evaluation of standardized humor interventions (the Humor Habits Program; McGhee, 2010) might be fruitful for the context of palliative care as well.

Future research should use widely agreed definitions of humor and validated assessment instruments. Data from RCTs with humor interventions from different palliative care settings are needed. In addition, training interventions for palliative care teams would be useful, teaching them to use humor as a resource to prevent burnout, but also fostering an emotional atmosphere that allows patients to express humor in their interactions with staff. This would be an efficient way to introduce humor on a structural level with members of staff. By doing so, humor could be implemented in palliative care with a long-term perspective rather than within the restricted setting of a clinical trial. Providing this kind of evidence will allow humor interventions to become part of the palliative care toolbox, to help lightening the burden of patients, caregivers, and health care professionals.

## Author contributions

The study design and search strategy were conceived by LL-D, LR, and SH. LL-D performed the literature search and screened the search results. Publications were reviewed by LL-D and LR. The manuscript was prepared by LL-D with support from SH, WR, and LR. All authors critically reviewed and contributed to the manuscript and approved the final version.

### Conflict of interest statement

The authors declare that the research was conducted in the absence of any commercial or financial relationships that could be construed as a potential conflict of interest.
