# Supplementary material for: Humor Assessment and Interventions in Palliative Care: A Systematic Review
Source: Front Psychol. 2018 Jun 19;9:890. doi: 10.3389/fpsyg.2018.00890 (PMC6020769; doi:10.3389/fpsyg.2018.00890)
Supplement: Supplementary file 1 [file Table_1.DOCX]

| **Criteria for qualitative studies…** | | **Criteria for quantitative studies…** | |
| --- | --- | --- | --- |
|  | **n=** |  | **n=** |
| Experienced/trained interviewers/scientists? |  | Experienced/trained interviewers/scientists? |  |
| Interviewers/Scientists independent from (clinical) care team? |  | Interviewers/Scientists independent from (clinical) care team? |  |
| Methods used to identify a representative population of participants? |  | Methods used to identify a representative population of participants? |  |
| Survey conducted within 6 months of experiences with care/decision? |  | Survey conducted within 6 months of experiences with care/decision? |  |
| **Reliability & Validity** | | | |
| Reliability of data collection (e.g. structures)? |  | Reliability of data collection (e.g. guides)? |  |
| Data validity (e.g. pretests)? |  |  |  |
| Full range of effects on patients (+caregivers?) evaluated or specific effects? |  | Data validity (e.g. pretests)? |  |
| Theme saturation reached? |  |  |  |
| Verbatim answers recorded? |  | Number of participants specified needed to reach statistical significance? |  |
| Independent & blinded coding? |  |  |  |
| **Reporting of results** | | | |
| Response rate > 50%? |  | Response rate > 50%? |  |
| Patients’(caregivers’) characteristics?  (More than age & gender) |  | Target number of participants reached? |  |
|  |  | Sociodemographic characteristics typed? |  |
| Emotional, medical & organizational setting typed? |  | Financial/organizational/care aspects typed? |  |
|  |  | Caregiver´s benefit typed? |  |
|  |  | Numbers & percentage typed? |  |
| Results on professional caregivers typed? |  | Results for professional caregivers typed? |  |
| Informational needs/wishes/priorities typed |  | Informational needs/wishes/priorities typed |  |
| Possible confounders evaluated? (Small sample size not enough) |  | Possible confounders and/or limitation to data evaluated? |  |
| = Number of studies with fulfilled criterion | | | |
